# Anolis lizards as biocontrol agents in mainland and island agroecosystems

**DOI:** 10.1002/ece3.2806

**Published:** 2017-03-02

**Authors:** Ivan V. Monagan, Jonathan R. Morris, Alison R. Davis Rabosky, Ivette Perfecto, John Vandermeer

**Affiliations:** ^1^ Department of Ecology and Evolutionary BiologyUniversity of MichiganAnn ArborMIUSA; ^2^ Museum of ZoologyUniversity of MichiganAnn ArborMIUSA; ^3^ School of Natural Resources and the EnvironmentUniversity of MichiganAnn ArborMIUSA

**Keywords:** agriculture, *Anolis*, biodiversity, coffee berry borer, ecosystem services

## Abstract

Our knowledge of ecological interactions that bolster ecosystem function and productivity has broad applications to the management of agricultural systems. Studies suggest that the presence of generalist predators in agricultural landscapes leads to a decrease in the abundance of herbivorous pests, but our understanding of how these interactions vary across taxa and along gradients of management intensity and eco‐geographic space remains incomplete. In this study, we assessed the functional response and biocontrol potential of a highly ubiquitous insectivore (lizards in the genus *Anolis*) on the world's most important coffee pest, the coffee berry borer (*Hypothalemus hampei*). We conducted field surveys and laboratory experiments to examine the impact of land‐use intensification on species richness and abundance of anoles and the capacity of anoles to reduce berry borer infestations in mainland and island coffee systems. Our results show that anoles significantly reduce coffee infestation rates in laboratory settings (Mexico, *p *= .03, *F *= 5.13 *df *= 1, 35; Puerto Rico, *p *= .014, *F *= 8.82, *df *= 1, 10) and are capable of consuming coffee berry borers in high abundance. Additionally, diversified agroecosystems bolster anole abundance, while high‐intensity practices, including the reduction of vegetation complexity and the application of agrochemicals were associated with reduced anole abundance. The results of this study provide supporting evidence of the positive impact of generalist predators on the control of crop pests in agricultural landscapes, and the role of diversified agroecosystems in sustaining both functionally diverse communities and crop production in tropical agroecosystems.

## 1 Introduction

The relationship between biodiversity and ecosystem function has received much attention due to growing concerns around the negative impacts of intensified land use. Empirical and theoretical studies suggest that biodiversity stabilizes ecosystem function, as referenced in the “insurance hypothesis,” whereby functional diversity acts as a buffer for ecosystem processes amidst environmental disturbance (Ives, Klug, & Gross, 2000; Yachi & Loreau, 1999). These principles have been applied broadly to the management of agricultural landscapes, which vary in both structural diversity and external inputs (Altieri, 1999; Perfecto, Vandermeer, Mas, & Pinto, 2005). Diversified agroecosystems that model native landscapes have been shown to function as reservoirs for local biodiversity (Fahrig et al., 2011; Tscharntke, Klein, Kruess, Steffan‐Dewenter, & Thies, 2005; Perfecto & Vandermeer, 2008) and suitable outlets for species dispersal among metapopulation communities (Vandermeer & Perfecto, 2007). Furthermore, increasing diversity can support ecosystem services that increase crop yield, such as the biological control of crop pests by natural enemies (Kremen & Miles, 2012; Vandermeer, Perfecto, & Philpott, 2010). Our understanding of how trophic interactions bolster ecosystem services such as biocontrol, and the response of relevant species to habitat modification may inform both socioeconomic and ecological goals of food security and biodiversity conservation.

The sustainable management of crop pests is an issue of increasing importance among farmers worldwide. In approximately 80 countries throughout the tropics (nearly 40% of all sovereign nations), coffee production is a leading agricultural commodity and the primary means of subsistence for nearly 20 million coffee‐growing households (Vega et al., 2015). The coffee berry borer (CBB), *Hypothenemus hampei*, is one of the most important and devastating coffee pests, inducing 60–90% reductions in coffee yields throughout many countries including, but not limited to, Mexico, Jamaica, Malaysia, and Tanzania (Benavides, Vega, Romero‐Severson, Bustillo, & Stuart, 2005). The destruction of the coffee berry occurs during the life cycle of *H. hampei;* wherein reproduction occurs within the fruit, the coffee seed is consumed by the brood (during stages of development), and adults emerge to disperse for oviposition in unoccupied berries (Brun, Stuart, Gaudichon, Aronstein, & French‐Constant, 1995; Vega et al., 2015). Several strategies have emerged to eliminate the berry borer, including agricultural intensification (Perfecto et al. 1996; Soto‐Pinto, Perfecto, & Caballero‐Nieto, 2002) and the application of insecticides (Brun et al., 1995). However, because the bulk of the organism's life cycle occurs within the fruit, topical pesticides are often ineffective (Damon 2000), and in cases where it is affected, CBB can quickly develop resistance to these chemicals (Vega, 2015).

Several mechanisms have been cited as promoting the top‐down control of herbivorous prey in ecological systems, with habitat complexity and predator diversity as highly relevant, especially to managed systems (Iverson et al., 2014; Philpott, Pardee, & Gonthier, 2012). A variety of naturally occurring biocontrol agents against the coffee berry borer have been documented, including ants (Gonthier, Ennis, Philpott, Vandermeer, & Perfecto, 2013; Larsen & Philpott, 2010; Morris, Vandermeer, & Perfecto, 2015; Perfecto & Vandermeer, 2006) and birds (Johnson, Kellermann, & Stercho, 2010; Karp, Mendenhall et al., 2013; Karp, Moeller, & Frishkoff, 2013). In an experiment conducted by Johnson et al. (2010), coffee plants excluded from foraging birds and bats had substantially higher coffee berry borer infestations. Furthermore, bird and bat densities were greatest in more structurally diverse farms.

Arboreal lizards in the genus *Anolis* (Iguanidae) are highly ubiquitous insectivores throughout the New World tropics and reach the highest population densities of any lizard in the world (Schoener & Schoener, 1980; Vitt, Avila‐Pires, Zani, Sartorius, & Espósito, 2003). Anoles drive the top‐down regulation of arthropod communities due to their dominant presence, especially in island ecosystems (Spiller & Schoener, 1990). Despite the high abundance and distribution of anoles, very few studies have addressed their functional role as predators in agroecosystems (Borkhataria, Collazo, & Groom, 2006; Borkhataria, Collazo, Groom, & Jordan‐Garcia, 2012). An exclusion experiment in Puerto Rican shade coffee found a negative impact of anoles on select herbivorous pests (Borkhataria 2006), while studies of anoles in natural systems indicate diets dominated by arthropods including ants (Huang, Norval, Wei, & Tso, 2008; Vitt et al., 2003), spiders (Hodge, 1999; Pacala & Roughgarden, 1984; Vitt et al., 2003), and beetles (Simmonds, 1958; Wolcott, 1923). Simmonds (1958) provides evidence that anoles function as biological control against scale insects in Bermuda, while also consuming a variety of small insect prey (*e.g*., ants) in large quantities. Whether or not anoles are important predators of the coffee berry borer, however, remains unknown.

**Figure 1:**
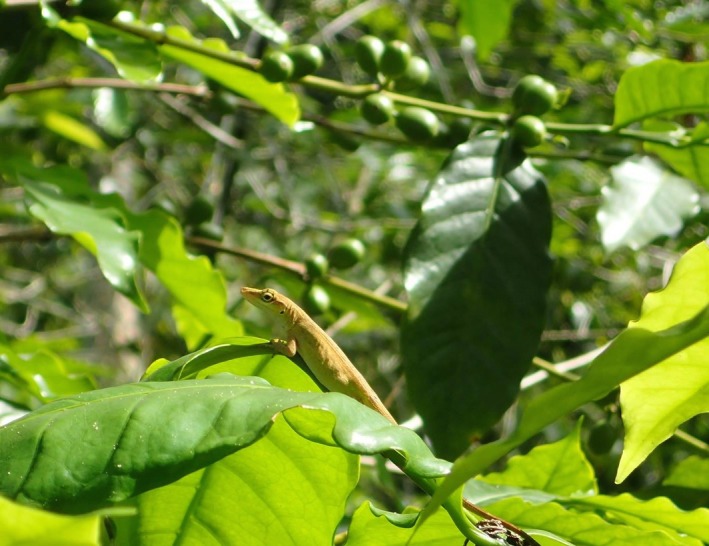
Photograph of an adult Mexican anole, *Anolis sericeus,* perching in a coffee shrub

*Anolis* lizards have been used broadly as a model group for the study of trait diversification and biotic interactions along environmental gradients (Losos, 2009). Their application to biocontrol appears most relevant due to an opportunistic feeding strategy, allowing individuals to monopolize on aggregates of prey (e.g., colonies of ants and termites) (Barbor 1930; personal observation). Comparative studies on the effects of anole presence and absence in island ecosystems show a negative correlation between the presence of anoles and plant damage via the reduction of herbivorous insect pests (Pacala & Roughgarden, 1984). Additionally, the ability of anoles to exploit vertical niche space, including coffee bushes (Figure 1), may bolster their capacity to serve as a front line of defense against most insect pests, particularly during outbreaks.

Differences in the evolutionary history and complexity of mainland and island lizard assemblages have led to novel ecological differences among mainland and island *Anolis* populations (Andrews, 1979). The adaptive radiation of Caribbean anoles into distinct ecomorphs that partition vertical and thermal niche space (Langerhans, Knouft, & Losos, 2006) is a feature that may have profound impacts on pest control services along complementary gradients of intensification. Our knowledge of how critical abiotic features such as temperature (Hertz, 1992; Huey, 1982) and light (Leal & Fleishman, 2002) generally influence species presence along gradients of land use remains elusive. Mainland studies of anoles in agroecosystems show both an increase (Mexico; Urbina‐Cardona, Olivares‐Pérez, & Reynoso, 2006) and a decrease (Mexico; Suazo‐Ortuno, Alvarado‐Diaz, & Martinez‐Ramos, 2008) in richness and abundance with agricultural intensification, while studies in island systems also show a negative response to increasing disturbance (Dominican Republic; Glor, Flecker, Benard, & Power, 2001) and a positive response of abundance in shifts from shade to open sun habitats (Puerto Rico; Borkhataria et al., 2012). The lack of comparable land‐use types and intensity metrics has made inferring underlying mechanisms that drive these differences difficult.

The coffee agroforestry systems of Latin America have been used broadly as a model for understanding the effects of land‐use intensification on biodiversity (Perfecto, Vandermeer, & Philpott, 2014). Coffee is generally grown along a gradient of vegetation complexity and land‐use intensity, including reduced canopy cover, reduced vegetative diversity, and chemical inputs (Moguel & Toledo, 1999). This important feature of coffee production, in addition to the well‐known ecological and biogeographic dynamics of anoles, makes them a model system and taxon for studying the role of diversity and ecological complexity in biological control.

In this study, we conducted an experimental and field‐based assessment of the potential for *Anolis* lizards to reduce coffee berry borer (CBB) infestations in regions of naturally high anole abundance (the Caribbean) and low anole abundance (Mainland Mexico). We investigated patterns of anole abundance and richness along a comparable gradient of agricultural intensification in the mainland and Caribbean coffee‐growing regions of Mexico and Puerto Rico to test the hypotheses that (1) anoles, as opportunistic and generalist predators, function to reduce CBB infestations in both mainland and Caribbean agroecosystems, and (2) differences in mainland and island community structure will result in a nonuniform response in anole richness and abundance to complementary forms of agricultural intensification, due to the stabilizing force of greater functional diversity in island ecosystems.

This study of generalist insectivores that exist in agricultural landscapes and are highly abundant across eco‐geographic space may help to identify land‐use practices that impact the ecosystem service of biocontrol. Furthermore, this approach has broad implications for understanding how phenomena such as adaptive radiation among potentially relevant species may provide ecological and evolutionary insights on the role of preadapted functional traits that shape community resilience to human‐modified environments.

## 2 Methods

### 2.1 Study sites

**Figure 2:**
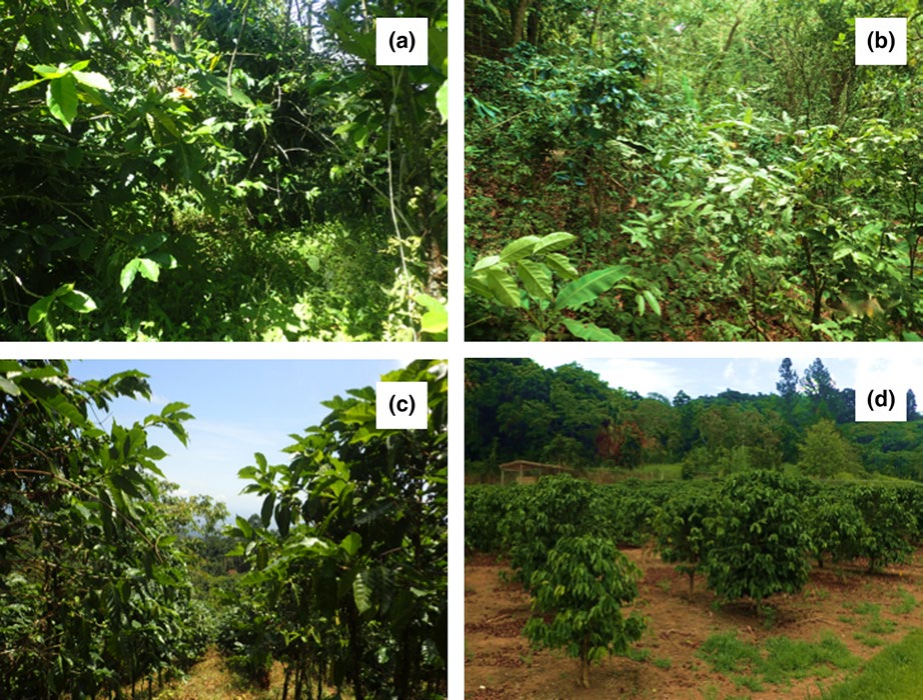
Representative photographs of diversified shade coffee in Mexico (a), diversified shade coffee in Puerto Rico (b), intensive sun coffee in Mexico (c), and intensive sun coffee in Puerto Rico (d)

Field surveys were conducted in the Soconusco region of Chiapas, Mexico, and the Puerto Rican municipalities of Orocovis and Adjuntas during the months of June and July 2015, respectively. The coffee‐growing landscape in Mexico is characterized by large farms (~300 hectares) with remnant patches of tropical evergreen forests making up approximately 6% of the 52‐km^2^ area covered. A total of twenty‐three 50 × 25 m sampling sites were surveyed along a gradient of shaded canopy cover and intensity (Figure 2a,c), within an altitudinal range of ~1,100–1,200 m above sea level. In Puerto Rico, coffee farms were more distinctly divided into shaded and unshaded management regimes and notably smaller in size (~1–6 ha per farm; Figure 2b,d). Survey sites were selected in a similar landscape of high‐altitude (550–730 m asl) farms within a matrix of tropical forest. A total of six 50 × 25 m plots were sampled along a gradient of canopy cover and intensity analogous to that of Mexico.

### 2.2 Field survey methods

Visual encounter survey methods were used to survey for all lizards in each 1,250‐m^2^ plot. Each plot was surveyed by walking each row of coffee and carefully inspecting each bush and surrounding vegetation up to three vertical meters for the presence or absence of anoles. Surveys took place between 10:00 and 15:00 hr because anoles were most active during this time (personal observation). Survey time for each plot was measured as the total time required for a single person survey effort per row divided by the total number of persons involved. In each plot, the total number of individuals encountered was recorded and each individual was identified to species.

Following lizard surveys, we took four vertical digital canopy cover photographs (DCP; adapted from Chianucci, Cutini, Corona, & Puletti, 2014) along a grid of sixteen localities per 32 m^2^ within the 1,250‐m^2^ plot area. Digital cover photography is a robust and time‐effective alternative to handheld densiometers, which is another common method of characterizing canopies (Chianucci et al., 2014). All photographs were taken using a point‐and‐shoot digital camera (Olympus Stylus Tough TG‐4) using the following settings: Photographic lens was set to F2, aperture priority, ISO 100, automatic focus, and exposure. In the field, photographs were taken at a height of approximately 1.5 m. Images were collected between the hours of 10 a.m. and 3 p.m. All photographs for each point along the survey grid were analyzed and averaged into a single value for each plot.

### 2.3 Site classification

Each survey plot was scored according to five major qualitative characteristics associated with both agricultural intensification and lizard abundance common to both Mexico and Puerto Rico (Figure 2). Characteristics analyzed included road‐induced edge effects (R), the application of pesticides (P), average coffee height (above or below 1.5 m) (S), and percent canopy cover (C). An agricultural intensity index (AII) was generated using the following equation:

AII=(R+P+S)−C

R (roadside), P (agrochemicals), and S (height) are binary variables given a value of 1 for presence and 0 for absence. Plots that were present approximately one meter from a vehicle path or road were assigned a value of 1, whereas interior plots were assigned a zero value. Agrochemical application was determined via land owner inquiry regarding the history and current use of agrochemicals. The existent use of agrochemicals was assigned an intensity value of 1. The agrochemical varieties and brands used were not recorded. Reduced coffee height (<1.5 M) was quantified as more intense and received a value of 1, while larger coffee (>1.5 M) received a zero value. Percent canopy cover (C) was included as a raw cover value in decimal form. Index values for each region range between −1 and 2, with a value of 2 corresponding to greatest intensity (Perfecto et al., 2005).

### 2.4 Laboratory experiments

#### 2.4.1 Field collection and husbandry

For laboratory experiments in both Mexico and Puerto Rico, lizards were collected by noose or butterfly net from a single coffee farm in each region that was characterized by dense canopy cover and absence of pesticide application. Individuals were collected after completing field surveys and on plots with an AII score ranging from 0 to 0.5. These site characteristics were chosen in order to reduce the potential for gross fitness differences among individuals. Upon collection, each individual lizard was assigned a number and GPS coordinate at the site of capture. A series of morphological measurements were collected, including snout–vent length and sex. Lizards were sexed using noninvasive transillumination technique described by Davis and Leavitt (2007), whereby a small LED light was positioned at the tail base (contralateral to the cloaca) to illuminate the presence or absence of male hemipenes. Individuals were also inspected for the presence or absence of a dewlap, which can also indicate sex in adults. Anoles of 38–45 mm snout–vent length were used for each laboratory experiments because they were the most frequently encountered size class for both Mexico and Puerto Rico.

#### 2.4.2 Infestation reduction experiment

**Figure 3:**
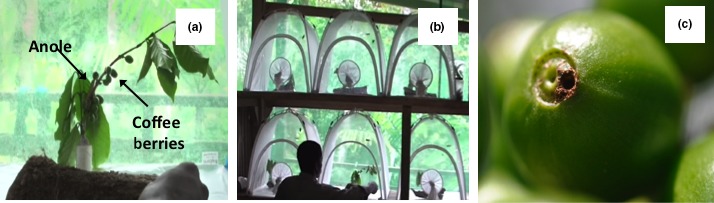
Laboratory setup for the experimental assessment of CBB infestation reduction. Each individual anole was paired with a single coffee branch per enclosure tent (a) and all enclosure tents were housed in a semi‐outdoor laboratory with natural sunlight and ambient conditions (b). (c) is a representative photograph of CBB entry holes used to assess coffee berry infestation

The infestation reduction potential of anoles was assessed by housing an individual anole in a 60 × 60 × 60 cm BugDorm© experimental mesh tent containing a single coffee branch (Figure 3a). Experiments were conducted in a semi‐outdoor laboratory with a single mesh screen wall that provided a natural photoperiod and ambient temperatures sufficient for natural feeding activities for the lizards. Branches with bored fruits were selected from the field to ensure that the berries were ripe enough for infestation by the berry borer. All bored berries and insects were removed from each selected branch before the start of the experiment, with twenty fruits and multiple leaves left remaining on each branch. Individual branches were positioned vertically in 35‐mm plastic canisters filled with water (Figure 3b). The top of each canister and branch based was wrapped in Parafilm© plastic to prevent CBB mortality. Each branch was then placed in the center of an inverted plastic bowl for vertical orientation and covered by a strip of bark. Bark was used to increase basking area and allow the anole to move freely from the coffee branch to the base of the enclosure.

Prior to each trial, a solitary lizard was housed in each BugDorm for a minimum of 24 hr to allow them to acclimate (Sanger, Hime, Johnson, Diani, & Losos, 2008). Berry borers were collected from infested fruits and removed carefully by splitting the fruit body and separating individual beetles from the plant material. At the start of each trial, twenty adult female berry borers were placed near the center of each branch using a coffee leaf as a platform. The platform was kept stable until all CBB had dispersed onto the branch. Following the 24‐hr trial window, each coffee branch was removed and the total number of infected berries per branch (number of berries with at least one CBB hole) was recorded (Figure 3c). Each lizard was returned to the original location of capture after the experiment was completed.

#### 2.4.3 Functional response

To assess the consumption potential of anoles, individuals were housed in 9.1‐kg aerated plastic terrariums with coffee leaves as substrate for 24 hr prior to the start of each trial. Terrariums were coated with fluon (Insect‐a‐Slip, BioQuip, CA) at the top to prevent CBB from escaping. Cardboard barriers were placed in between terraria to prevent visibility among individuals. Terrarium holes were created using a small 16‐gauge pin needle to ensure airflow, but to prevent the beetles from escaping.

Adult female berry borers were obtained from infested berries collected in the field and placed into separate glass vials hours prior to the start of each experiment. CBB were housed for no longer than 24 hr to ensure borer efficacy. Berry borers were placed in the terrariums between the hours of 9 and 10 a.m. Each trial lasted for twelve hours, after which lizards were removed from each container and all unconsumed beetles were recorded. All remaining beetles were euthanized following each experiment. Morphometric measurements taken for each individual lizard included snout–vent length, head width, head length, tail length, front and hind limb length, in addition to sex, gravidity, and species.

### 2.5 Data analysis

#### 2.5.1 Field surveys

Canopy cover images were analyzed using a dot‐grid approach to estimate canopy cover for each sample location. Interpretation of digital cover photographs using a transparent dot‐grid overlay is a standard technique well suited for estimating canopy cover (Nowak et al. 1996).

An analysis of variance (ANOVA) test was used to find statistical significance between total abundance and region. Linear regressions were used to examine the effect of canopy cover on total lizard abundance per region. We used generalized linear mixed models (GLMMs) to examine the relative importance habitat variables on abundance.

#### 2.5.2 Laboratory experiments

Generalized linear models (GLMs) were used to account for covariates in differences between consumption patterns (functional response) and berry borer infestation rates between treatments with and without anoles. Differences in coffee borer infestation rates were analyzed with an ANOVA.

Linear and nonlinear models were used to fit the CBB consumption data for Mexico, Puerto Rico, and the combined data set to the following functional response models as outlined by Holling (1959, 1965):

Type I:P=aN

$$\text{Type II:}\;P\;=\;\frac{aN}{1\;+\;hN}$$

$$\text{Type III:}\;P\;=\;\frac{aN^{2}}{1\;+\;hN^{2}}$$

where *P* is the total number of coffee berry borers consumed, *N* is prey density (total number of CBB offered), *a* is attack rate, and *h* is handling time. Attack rate and handling time were not measured directly in this study and were included as constants in the model. The AIC value of each model was used to assess performance, with the lowest value indicating the best fit to the data. All statistical tests were performed in R v3.2.3, and significance was assessed at a *p* value ≤.05 (R Core Team, 2015).

## 3 Results

### 3.1 Infestation reduction potential and functional response

**Figure 4:**
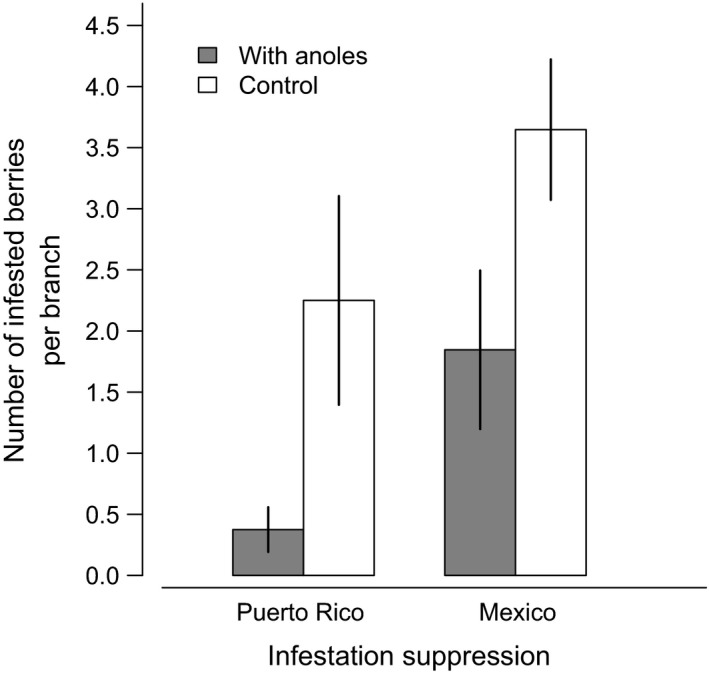
Mean number of coffee berries infested by the coffee berry borer (±1SE) in the presence and absence of *Anolis* lizards in laboratory settings

In laboratory settings, individual anoles reduced coffee berry borer infestations by an average of 49% in Mexico (*p *= .03*, F *= 5.13, *df *= 1, 35) and 83% in Puerto Rico (*p *= .019, *F *= 8.82, *df *= 1, 10; Figure 4). The effects of sex and gravidity on reduction potential were nonsignificant (*p *> .05).

**Figure 5:**
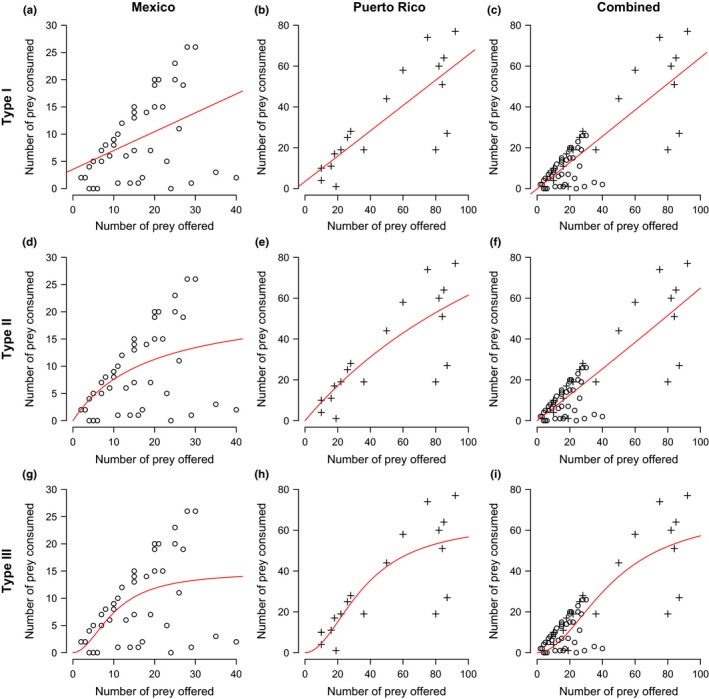
Functional response of anole predation on variations in coffee berry borer abundance in laboratory settings. Figures show data fitted to Type I (a‐c), Type II (d‐f), and Type III (g‐i) functional response curves for Mexico, Puerto, and Combined data sets

Manipulations of prey density reveal a type III functional response by anoles for data from Mexico and Puerto Rico (Figure 5). The combined data, however, reveal indistinguishable differences between the type I and type II AIC values (Table 1). Results from a generalized linear model suggest that gravidity, snout–vent length, species, and region are nonsignificant effects on consumption potential (*p* > .05; Table 2).

**Table 1:** AIC values for type I, II, and II functional response model fit to the given data for Mexico, Puerto Rico, and combined

Functional Resp.	AIC Values		
	Mexico	Puerto Rico	Combined
Type I	293.54	167.79	475.38
Type II	290.19	167.17	475.35
Type III	288.76	166.72	479.53

**Table 2:** Results of a generalized linear mixed model testing the effects of canopy cover, agrochemicals, edge effects, and coffee height on anole abundance in plots in Mexico and Puerto Rico. Asterisks denote degree of significance. * indicates < .05, ** indicates < .01

Variable	Fixed Effects	Estimate	*SE*	*Z*	Pr(>|z|)	Random effects	Variance	*SD*
Region: Mexico								
Abundance	Intercept	2.644	1.666	1.587	0.113	Plot	0.351	0.592
	Cover	−1.289	2.324	−0.555	0.579			
	Agrochem	−3.671	1.072	−3.424	0.006**			
	Road	0.2655	0.4727	0.562	0.574			
	Height	−1.706	0.703	−2.427	0.015*			
Region: Puerto Rico								
Abundance	Intercept	2.104	0.894	2.353	0.0186*	Plot	0.656	0.81
	Cover	3.183	1.149	2.769	0.005**			
	Road	−0.951	0.737	−1.289	0.197			

### 3.2 Environmental predictors of abundance and species presence

**Figure 6:**
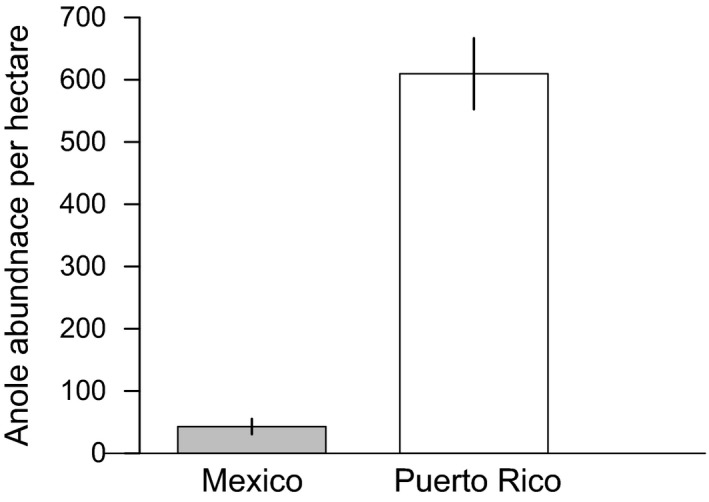
Average abundance of anoles per hectare in Mexico (*n* = 42.9 ± 12.56) and Puerto Rico (*n* = 609.6 ± 57.26) from plots where anoles were present

The average abundance of anoles on all coffee plots containing at least one individual was approximately twelve times greater in Puerto Rico than in Mexico (Figure 6). Anoles were the only lizard genus found on farms in Puerto Rico (five species total), while the two species of anole known on farms in Mexico were present along with a single species of *Amieva* and an unidentified species in the genus *Mabuya* (Table 3). In Mexico, a single species of anole was dominant throughout the study area (*A. dollfusianus*), while the less dominant species were present only in plots with reduced shade cover ranging from 50 to 75% cover (Table 3). Both species in Mexico also favored plots with coffee plants that were on average greater than 1.5 m in height.

**Table 3:** Average species abundance per characteristic of habitat intensity in Mexico and Puerto Rico

	% Canopy cover		Agrochemical use		Roadside plot		Coffee height	
	50–75%	75–100%	Yes	No	Yes	No	<1.5 M	>1.5 M
Mexico								
* Anolis dollfusianus*	6.8	8	0.08	5.18	7.3	6.7	1.4	8.3
* Anolis sericeus*	1.6	0	0.08	0.09	1	1.7	0	1
* Amieva amieva*	0.8	0	0.25	0.27	0.7	1	0.2	0.66
* Scincidae spp*.	3.6	0	0.17	2	3.3	2.7	0.8	2.66
Total	12.8	8	0.58	7.54	12.3	12.1	2.4	12.62

	% Canopy cover		Agrochemical use		Roadside plot		Coffee height	
	0–25%	75–100%	Yes	No	Yes	No	<1.5 M	>1.5 M
Puerto Rico								
* *Trunk–Ground								
* Anolis gundlachi*	1	43.5	0	46.4	7	68	N/A	46.4
* Anolis cristatellus*	30	7	0	12.4	26.5	0	N/A	12.4
* *Trunk–Crown								
* Anolis stratulus*	18	1.5	0	4.8	1.5	0	N/A	4.8
* Anolis evermanni*	1	2.5	0	5.8	2.25	9	N/A	5.8
* *Grass–Bush								
* Anolis krugi*	2	1	0	1.4	0.75	0	N/A	N/A
Total	52	55.5	0	70.8	38	77	N/A	69.4

Coffee plantations in Puerto Rico were generally dominated by a single species in plots with high shade (*A. gundlachi*) and plots with low shade (*A. cristatellus*; Table 3). The less dominant species, *A. evermanni* and *A. stratulus*, also occurred more frequently in shade or sun plots, respectively. All four species generally occurred together when plots were positioned along a road or habitat edge.

**Figure 7:**
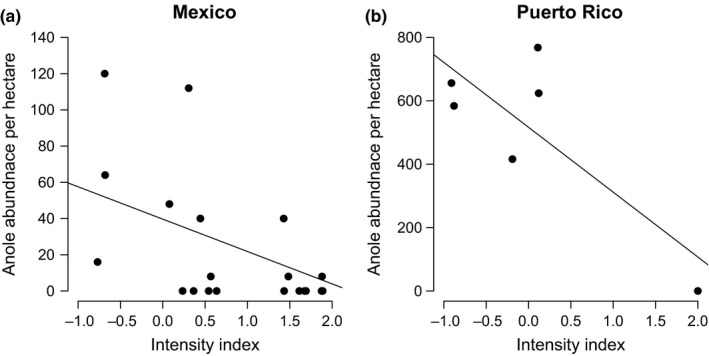
(a) Variation in anole abundance along a gradient of intensity in Mexico (*R* ^*2*^ = .278, *p *= .006) and (b) Puerto Rico (*R* ^2^ = .539, *p* = .059)

Along a gradient of increasing agricultural intensity, both Mexican and Puerto Rican anole abundance decreased significantly (Mexico: *R* ^*2*^ = .278, *F *= 9.48, *df *= 1, 21, *p* = .006; Puerto Rico: *R* ^*2*^ = .539, *F *= 6.85, *df *= 1, 4, *p* = .059; Figure 7). In Mexico, only 11 of 23 surveyed plots contained anoles, while 6 of the 11 were present at the lowest index values ranging from −1.0 to 0.5. In Puerto Rico, the greatest abundance of anoles was not *found* at the lowest intensity value, but *abundance* did show a linear decrease with increasing intensity. This trend appears to be driven by a single plot with zero anoles. The generalized linear mixed model testing the effects of canopy cover, agrochemicals, edge effects, and coffee height on anole abundance in plots in Mexico and Puerto Rico revealed significant effects of coffee height (positive) (*p* = .015, *Z *= −2.43; Table 2) and agrochemical application (negative) (*p* < .05, *Z *= −3.42; Table 2) on abundance in Mexico and significant effects of canopy cover (positive) (*p* = .005, *Z *= 2.77; Table 2) on abundance in Puerto Rico. In both regions, the application of agrochemicals had a deleterious effect on anole abundance (Table 3), but lack of necessary replication of pesticide plots in Puerto Rico (*N* = 1) prevented this parameter from being used in the model.

## 4 Discussion

### 4.1 The biocontrol potential of *Anolis* lizards on CBB

The results of this study are the first to provide evidence that anoles are capable of both consuming the coffee berry borer in high numbers (Figure 5) and significantly reducing CBB infestations in the laboratory settings (Figure 4). These results, combined with our field survey data showing that anole abundance is bolstered by reduced agricultural intensification (Figure 7), suggest that anoles may be important biocontrol agents in diversified coffee landscapes, particularly in regions such as Puerto Rico where they are naturally more abundant. Furthermore, these results support several theoretical and field‐based studies suggesting that pest control services decline significantly when generalist predators are removed from coffee agricultural landscapes (Faria, Umbanhowar, & McCann, 2008; Karp, Mendenhall et al., 2013; Karp, Moeller, & Frishkoff, 2013; Perfecto et al., 2004).

Predation rates by lizards are generally determined by many other factors, including prey diversity, predator size, and environmental conditions such as habitat diversity and seasonality (*e.g*., Angilletta, 2001; Pitt & Ritchie, 2002). This study was conducted during the egg laying season for Mexican anoles and during the period of low berry borer dispersal and abundance for both regions (Sponagel, 1994), so the functional response of anoles to coffee berry borer abundances may be different in field settings at other times of the year. Realistic estimates of reduction potential would be most robust for experiments conducted in natural conditions, with natural variation in ecological factors like structural complexity and prey diversity.

Results from the functional response experiment imply that more data are necessary to infer a functional response curve for the combined data set or that the data better fit an alternative model (Table 1). AIC values for Puerto Rico show negligible differences between each functional response type, suggesting that more data are needed to infer a satiation point. This result also suggests that the combined data set significance may have been driven primarily by the Mexico data. Overall, however, the high consumption results from this study are concordant with several studies showing that anoles consume large numbers of insects that may have been otherwise assumed too small relative to lizard body size to reflect an important diet component (Simmonds, 1958). Ultimately, the behavior of the coffee berry borer in field settings, with added variables like habitat variability and coconsumption of alternative prey, may provide more realistic estimates of functional response for this genus.

### 4.2 Adaptive radiation as a predictor of disturbance tolerance

Previous studies documenting the effects of agricultural intensification corroborate the results of this study that shifts from diverse ecosystems to intensified agricultural landscapes have negative effects on the functional characteristics of anole communities such as abundance, diversity, and use of vertical plant space (Borkhataria et al., 2012; Glor et al., 2001). This study additionally illustrates the deleterious effects of agrochemical use in Mexico and Puerto Rico, and the significant role of dissimilar habitat variables on abundance in each region (coffee height in Mexico, canopy cover in Puerto Rico). For plots that included agrochemical applications, lizards of both regions were virtually eliminated, potentially because of reduced prey abundance, or perhaps through direct bioaccumulation of toxic substances (Mann, Sánchez‐Hernández, Serra, & Soares, 2007).

The sympatric occurrence of distinct ecomorphs in the Puerto Rican coffee farms is posited here as the underlying mechanism leading to higher abundances at intermediate levels of intensity among island anoles (Figure 7). Although lizards are generally assumed to favor basking sites and open habitats for thermoregulation, several mechanisms may account for a dissimilar response between island and mainland taxa. Anoles have been shown to be either thermoregulators, species that actively select favorable microhabitats, or thermoconformers, species that adopt ambient temperatures (Losos, 2009). Comparative studies of the Puerto Rican anoles *A. gundlachi* and *A. cristatellus* reveal that *A. gundlachi* functions as a thermoconformer adapted to cooler environments and *A. cristatellus* as a thermoregulator tolerant of warmer conditions (Hertz, 1992; Rogowitz, 1996). Our results corroborate this finding by showing increased *A. gundlachi* abundance in interior plots with high shade (Table 3), whereas *A. cristatellus* was most abundant in plots with the least amount of shade and along forest edges with reduced cover (Table 3). *Anolis stratulus* was also shown to share trends similar to that of *A. cristatellus*, supporting findings by Borkhataria et al. (2012), who showed congruent relationships to sun and shade dominance among *A. gundlachi* and *A. cristatellus*. Mainland anole species have been reported to avoid the costs of thermoregulation by selecting for environments that are relatively warmer (Vitt, Sartorius, Avila‐Pires, & Espósito, 2001). The results of this study, however, suggest that mainland anoles respond more to shifts in structural diversity than to reduced cover or habitat edges.

A number of additional mechanisms may influence the reduction of anole diversity in sun and pesticide plots between the two regions. As discussed previously, the life‐history characteristics of Caribbean island and mainland anoles are understood to be fundamentally different. Anole communities within the Caribbean are limited by food resources due to high interspecific competition, whereas mainland anoles are generally limited by relatively greater levels of predation (Andrews, 1979). Andrews (1979) additionally references mainland anoles as having lower survivorship and lower food intake (via less time foraging). Although mainland anoles with low abundances are not predicted to have as great of an ecosystem impact on the insect community as island anoles, they are likely more vulnerable to changes in prey availability, structural diversity, and chemical inputs.

### 4.3 Implications for management and conservation

The results of this study imply that the geographic location and local environmental settings where human disturbance takes place are both important factors that must be considered when managing at‐risk species. This research suggests that the structural diversification of coffee farms functions as a benefit both to farmers, by providing the insurance of predatory diversity against pest outbreaks, and to biodiversity, by providing a hospitable landscape for persistence and dispersal.

In the island agroecosystems of Puerto Rico, the loss of anole biocontrol services is buffered by greater functional diversity and overall abundance, relative to Mexico, implying that islands of the greater Antilles are more equipped to respond to disturbance at the genus level. Shade‐adapted ectotherms such as *Anolis gundlachi* in Puerto Rico, however, will likely be isolated in forested habitat islands as the result of an increasing move toward sun coffee and deforestation, and they may be at greater extinction risk relative to species that are more tolerant to the higher temperatures experienced in more intensely managed farms (Frishkoff, Hadly, & Daily, 2015). A study of mainland anoles by Pounds, Fogden, and Campbell (1999) suggested that mainland anole abundance decreases linearly in response to increasing environmental temperatures. Such declines are predicted to be further exacerbated amidst intensified agricultural landscapes and increasing global temperatures (Deutsch et al., 2008).

In conclusion, the evidence presented in this study showing that anoles reduce pest infestation potential and are adversely effected by land‐use intensification has important implications for the management of agricultural landscapes to maintain ecosystem services such as biological control. This understanding adds to a growing body of evidence suggesting that win‐win solutions are possible in agriculture, helping both to conserve biodiversity and to promote the sustainable production of food to meet society's needs.

## Conflict of Interest

None declared.
